# The Mediating Role of Early Maladaptive Schemas in the Relation between Co-Rumination and Depression in Young Adults

**DOI:** 10.1371/journal.pone.0140177

**Published:** 2015-10-21

**Authors:** Michela Balsamo, Leonardo Carlucci, Maria Rita Sergi, Karla Klein Murdock, Aristide Saggino

**Affiliations:** 1 Department of Psychological, Health and Territorial Sciences, “G. d’Annunzio” University, Chieti, Italy; 2 Department of Psychology, Washington and Lee University, Lexington, Virginia, United State of America; Shanghai Mental Health Center, Shanghai Jiao Tong University School of Medicine, CHINA

## Abstract

Research on co-rumination has investigated its relationship with internalizing symptoms, but few studies have addressed underlying maladaptive cognitive-affective processes that may play an important role in the maintenance of this relation. This study examines if Young’s schema domains mediate the relation between co-rumination and depression in a community sample of non-clinical young adults. Participants completed the Co-Rumination Questionnaire, Young Schema Questionnaire-L3, and Teate Depression Inventory. Correlations and path analysis were calculated for the full sample and separately by gender. The schema domains of Overvigilance/Inhibition and Other-Directedness fully mediated the relation between co-rumination and depression. When analyses were performed separately for males and females, mediation persisted only for females. Findings suggest that among young women, co-rumination with a friend may be associated with depressive symptoms because of its activation of specific maladaptive cognitive schemas. Better understanding of the content and processes underpinning co-rumination may have important implications for the prevention and treatment of depression.

## Introduction

Co-rumination involves the tendency to engage with another person in extensive, negatively focused discussions in which one's reactions to ongoing problems are repeated and rehashed [1]. For instance, friends may repeatedly discuss a fight between a girl and her boyfriend, dissecting the exchange from every angle and dwelling on the negative implications and feelings triggered by it. In a similar fashion, co-ruminating peers may actively encourage one another to continue talking, rehashing, speculating about, and dwelling on a perceived slight from a peer, such as not being invited to a party.

### Co-rumination as a Risk Factor for Psychosocial Distress

Although co-rumination has been linked to positive perceptions of friendship quality in youths [1,2], it also has been associated with increased anxiety and depressive symptoms [1–6]. Most recent studies have investigated specific forms of co-rumination and their associations with internalizing symptoms [5–9].

However, the literature on co-rumination has begun to illuminate contexts and conditions that drive and/or exacerbate its association with psychosocial distress. For instance, it appears that exposure to interpersonal stress can shape the implications of co-ruminative behavior for mental health [5,8,10]. The salient role of interpersonal stress has been reinforced by Nicolai, Laney, and Mezulis [11], who found that co-rumination about interpersonal stressors, but not other types of stressors, was associated with depression. Several other studies have attempted to clarify how co-ruminative *content* is associated with specific aspects of well-being. For example, Starr and Davila [6] found a significant association between co-rumination and increased depression among adolescent girls when co-rumination focused on a potentially distressing topic, such as adolescent romantic experiences. Moreover, adolescents who experience high levels of social anxiety have been found to be more likely to engage in co-ruminative discussions about social events and their social performance with their best friend [11,12], suggesting that social cues concerning others' perceptions may serve as triggers for co-rumination.

### Co-rumination, Cognitive Processes, and Depressed Mood

Although the precise mechanisms through which co-rumination increases psychological risk are not known, cognitive processes may be fruitful targets of investigation. Unfortunately, few studies have addressed underlying maladaptive cognitive processes that may account for the relationship of co-rumination with depression symptoms. Beck [13] noted that the origin of depression might be at the schematic level of cognition, and Young [14] revised the schema concept, emphasizing early maladaptive schemas (EMSs) as key structures in the development of psychopathology. EMSs are defined as information processing structures concerning beliefs about oneself and one’s relationship with others and the world, which are developed during childhood and further established in peer relations during adolescence [15]. There is abundant evidence showing that schemas are related to symptoms of psychopathology, such as depression in adolescents [16] and adulthood [17].

To date, 18 EMSs have been identified in asymptomatic populations and shown to be particularly distorted, rigid, and dysfunctional in symptomatic individuals [14]. Thus, Young’s schema theory might constitute a valuable framework to understand psychopathology in young adults.

The current study explored the mediating role that schema domains may play in the relation between co-rumination and symptoms of depression among non-clinical young adults. We hypothesized that the significant statistical relationship between self-reported co-rumination and concurrent depression would disappear after controlling for specific maladaptive cognitive schemas, suggesting that co-rumination contributes to depression due to the activation of these particular cognitive processes.

Mediational models investigate how a third variable affects the relation between a predictor and an outcome variable. A newer application of the mediation approach is in prevention and treatment research, where interventions are designed to change the outcome of interest by targeting mediating variables that are hypothesized to be causally associated with the outcome [18]. Therefore, a better understanding of processes underpinning co-rumination may have important implications for prevention and treatment development. For example, college students considering transferring out of their university often engage in co-rumination [19]. Therefore, deeper knowledge about co-rumination including understanding the conditions under which intensive discussion of problems becomes harmful could help educators to support students who are contemplating this important life decision by engaging in alternative strategies focused on problem solving [20].

### The Current Study

Given that associations between co-rumination and maladaptive cognitive schemas have not yet been empirically examined, the first goal of our study was to investigate these relationships utilizing the maladaptive cognitive schema domains identified by Young et al. [15]. We expected that co-rumination, with its persistent focus on problems, would trigger maladaptive cognitive schemas. Thus, we tested a preliminary cross-sectional hypothesis that these cognitions would mediate the relationship between co-rumination and depressive symptoms.

Another goal of this study was to explore possible gender differences in these processes. There is evidence that girls and women report higher levels of co-rumination than boys and men and that co-rumination predicts greater depressive symptoms in girls but not boys [2,5]. Furthermore, some evidence suggests that females demonstrate higher cognitive vulnerability for depression [21] and that cognitive vulnerabilities are more strongly linked to depression for females when compared to males [22]. Finally, the prevalence of depression is two times higher for females than for males [23,24]. Thus, we tested an exploratory hypothesis that gender differences would exist in the mediating role of maladaptive schema domains in the co-rumination—depression relation.

## Materials and Methods

### Participants

Eligible participants were between the ages of 18 and 38 years with the capacity to complete self-administered questionnaires. Exclusion criteria included marked cognitive impairment, a drug abuse disorder, psychotic symptoms, and major disorders of the central nervous system (e.g., Alzheimer's disease, Parkinson's disease, epilepsy).

Participants were 461 Italian subjects, including 254 females (55%) and 207 males (45%), of whom 362 (84.4%) were undergraduate students. The sample's mean age was 23.93 years (SD = 6.90 years; range = 18–36 years). The mean age for men was 25.91 years (SD = 7.70 years), and for women, 22.91 years (SD = 6.01 years). The mean level of education was 13.53 years (SD = 1.94 years). All subjects were white. The sample was recruited through advertisements (flyers, newspapers, and online ads) posted for established community groups (e.g., youth centers, church groups, university student associations). Study participants contributed voluntarily and anonymously; no honorarium was given for completing the assessments.

Written informed consent was obtained from all participants before starting the experiment, according to the Declaration of Helsinki. The study was approved by the Ethical Committee of the ‘‘G. d’Annunzio” University of Chieti-Pescara, Italy.

### Measures

All participants were administered the Italian versions of the Co-Rumination Questionnaire (CRQ), the Teate Depression Inventory (TDI), and the Young Schema Questionnaire Long Form, Third Edition (YSQ-L3). All respondents completed paper-and pencil versions of the questionnaires in a fixed order (a socio-demographic checklist, the YSQ L3, the CRQ, and the TDI) on site at established community groups. The protocol was administered by three licensed psychologists who had four hours of training wherein the objectives of the research, characteristics of the instruments administered, and information about common issues in psychological assessment among young adults was explained.

#### Co-Rumination Questionnaire (CRQ)

The CRQ includes 27 items designed to measure co-rumination between same-sex friends [1]. A sample question is, ‘‘If one of us has a problem, we will spend our time together talking about it, no matter what else we could do instead.” Participants were asked to think about the way they usually behave with their best or closest friend of the same gender. Each item was rated on a 5-point Likert scale ranging from 1 (not at all true) to 5 (really true). Total co-rumination scores were calculated by averaging participants’ ratings across the 27 items. The convergent validity of the co-rumination construct has been demonstrated previously with positive correlations to measures of both rumination (*r* = .46) and self-disclosure (*r* = .61) [1]. The CRQ has demonstrated moderate retest reliability at 6 months, *r* = .54 [2], and excellent internal consistency in non-clinical samples, alphas = .96–.97 [1,2,25]. Exploratory factor analysis indicated a single factor with all loadings over .45; Cronbach’s alpha was .96 [1]. In the present study the CRQ exhibited excellent internal consistency (alpha = .95) (Table 1).

**Table 1:** Descriptive Statistics, Reliability and Correlations between Co-Rumination with YSQ-L3 domains and Depression (*N* = 463).

	TDI	YSQ-L3					α	Mean	SD	Skewness	Kurtosis
		Disconnection/Rejection	Impaired Autonomy	Other-Directedness	Impaired Limits	Overvigilance/Inhibition					
CRQ	.104*	.180**	.265**	.319**	.209**	.322**	.95	2.73	.76	.07	-.57
TDI		.457**	.454**	.311**	.337**	.304**	.91	1.41	.59	.13	-.62
Disconnection/Rejection			.663**	.639**	.646**	.685**	.95	2.10	.58	.50	-.20
Impaired Autonomy				.685**	.552**	.615**	.94	1.90	.56	.76	.30
Other-Directedness					.524**	.665**	.92	2.45	.68	.38	.09
Impaired Limits						.697**	.87	2.40	.61	.11	-.21
Overvigilance/Inhibition							.93	2.52	.63	.32	-.16

* p < .05

** p < .01.

Note. CRQ = Co-Rumination Questionnaire; TDI = Teate Depression Inventory.

#### Young Schema Questionnaire- Long Form, Third Edition (YSQ-L3)

The YSQ-L3 is a 232-item self-report instrument designed to assess 18 EMSs [26]. Participants are asked to rate each statement on a 6-point Likert scale ranging from 1 (not true at all) to 6 (this describes me perfectly). Items are clustered by 18 scales and grouped into five domains, bringing together the EMSs that tend to develop together: Disconnection/Rejection (Abandonment, Mistrust/Abuse, Emotional Deprivation, Defectiveness/Shame, Social Isolation/Alienation); Impaired Autonomy/Performance (Dependence/Incompetence, Vulnerability to Harm or Illness, Enmeshment/Undeveloped Self, Failure); Impaired Limits (Entitlement/Grandiosity, Insufficient Self-Control/Self-Discipline); Other-Directedness (Subjugation, Self-Sacrifice, Approval-Seeking/Recognition-Seeking); and Overvigilance/Inhibition (Negativity/Pessimism, Emotional Inhibition, Unrelenting Standards/Hypercriticalness, Punitiveness). In keeping with the scoring key accompanying the YSQ-L3A, in the current study domain scores were obtained by averaging the scores of the scales included in the domain concerned. In all cases, a higher score reflects a more maladaptive, detrimental core belief. The subscales of previous versions of the YSQ have demonstrated high test–retest reliability and adequate internal consistency, as well as convergent and discriminant validity [15,27–30]. In the current sample, Cronbach’s alphas were .95 for Disconnection/Rejection, .94 for Impaired Autonomy/Performance, .92 for Impaired Limits, .87 for Other-Directedness, and .93 for Overvigilance/Inhibition (Table 1).

#### Teate Depression Inventory (TDI)

The TDI is a new 21-item self-report instrument designed to assess major depressive disorder [31], as specified by the latest editions of the *Diagnostic and Statistical Manual of Mental Disorders* (DSM-IV-TR, DSM-5; American Psychiatric Association) [32–33]. It was developed via Rasch logistic analysis of responses, within the framework of item response theory [34], in order to overcome inherent psychometric weaknesses of existing measures of depression, including the BDI-II [35]. Each item is rated on a five-point Likert-type scale, ranging from 0 (always) to 4 (never). A small but growing literature suggests that the TDI has strong psychometric properties in both clinical and non-clinical samples [31,36–40]. Three cut-off scores were recommended in terms of sensitivity, specificity, and classification accuracy for screening for varying levels (minimal, mild, moderate, and severe) of depression severity in a group of patients diagnosed with major depressive disorder [38]. In the present sample, Cronbach’s alpha was .91 (Table 1).

### Statistical Procedures

Zero-order correlation coefficients were calculated among co-rumination, schema domains as measured by the five subscales of the YSQ-L3, and depression. We considered as salient only absolute correlations equal to or greater than .30, which explains 9% or more of the variance, as the probability value is influenced by the number of subjects in the sample [41].

Our mediation hypotheses were tested using Baron and Kenny's causal-steps method [42]. A path analysis was calculated, using the SPSS macro MedText [43], to test if the subset of Young’s schema domains that was saliently related with co-rumination mediated between co-rumination and the TDI depression score.

Baron and Kenny’s [42] method utilizes a series of regression analyses to proceed through the steps of mediation testing. *Step 1* establishes the presence of a significant effect that may be mediated between the predictor / causal variable X and the criterion / outcome variable Y (path c, also called “total effect”). *Step 2* establishes that the predictor variable X affects the mediator / intervening / process variable, M; in the regression equation, M is used as the criterion (path a). *Step 3* establishes that the mediator variable M affects the outcome variable Y; in the regression equation, Y is used as the criterion variable and X and M as predictors (path b). It is not sufficient just to correlate the mediator with the outcome because the mediator and the outcome may be correlated; they may be both caused by the causal variable X. Thus, the causal variable X must be controlled in establishing the effect of the mediator on the outcome. *Step 4* establishes if M fully mediates the X-Y relationship; the effect of X on Y controlling for M (path c', also called “direct effect”) should be zero. The effects in both Steps 3 and 4 are estimated in the same equation.

In the case where, after controlling for M, the path from X to Y is reduced in absolute size but is still different from zero, then the test of Sobel [44] can be used to indicate *partial* mediation. This indirect effect occurs if the regression coefficient (β) for the mediator included in the model was significantly higher than the β for only Y regressed on X.

*Inconsistent mediation* or an *inconsistent mediator effect* is said to occur if the first step of mediation testing is not supported (i.e., path c, the total effect, is not statistically significant), and the direct effect tested in step 4 (path c’) is statistically significant but opposite in sign to the a and b paths revealed in steps 2 and 3 [18,45]. An inconsistent mediator acts like a suppressor variable: it is a third variable that increases the predictive validity of another variable by its inclusion in a regression equation. An inconsistent mediator effect would be present when the direct and mediated effect (c’) is larger than the total effect of X on Y (c). Then, the direct and indirect effects will tend to cancel each other out in the overall relationship (e.g., the Pearson’s r will be close to zero). However, including the third, inconsistent mediator variable in the regression equation will make the direct effect evident and at the same time demonstrate that the third variable mediated an opposite, inconsistent mediator effect.

Gender differences were examined in levels of co-rumination, salient cognitive schema domains, and depression. Gender differences in the mediational model were explored by performing the steps of mediation analysis for female and male subsamples separately.

## Results

### Descriptive Statistics

Zero-order correlation coefficients among total scores for the CRQ, five subscales of the YSQ-L3, and the TDI were calculated. These are presented in Table 1 along with descriptive statistics and internal consistency reliabilities of these measures. The univariate normality of all measures was illustrated with the largest skewness index of .76 and the largest kurtosis index of -.62 [46].

The relationship between co-rumination and depression was significant. The Young’s schema domains were found to have significant and positive correlations with co-rumination, ranging from .18 to .32 (Table 1). However, among the five Young’s domains, only Overvigilance/Inhibition and Other-Directedness were found to have salient correlations with the CRQ score (*r* = .322; p < .01; *r* = .319; p < .01, respectively). Depression was correlated at .30 and .31 (*p* < .01) with Overvigilance/Inhibition and Other-Directedness, respectively.

Concerning the influence of gender, there were significant differences on the total scores of the CRQ (M_males_ (SD) = 1.30 (.59), M_females_ (SD) = 1.50 (.57); *F*_1;459_ = 11.19; *p* < .001 and the TDI (M_males_ (SD) = 2.59 (.71), M_females_ (SD) = 2.83 (.78); *F*_1;459_ = 12.83; *p* < .001), and on. We also explored the data to determine whether the strength of the relationship between co-rumination and concurrent depression was different for young males and females. Gender differences were present in the strength of this association, as the relationship between co-rumination and depression was significant for females, *r*_(254)_ = .139, *p* = .038, while not significant for males, *r*_(207)_ = .027, *p* = .704.

### Mediation Analysis

**Fig 1:**
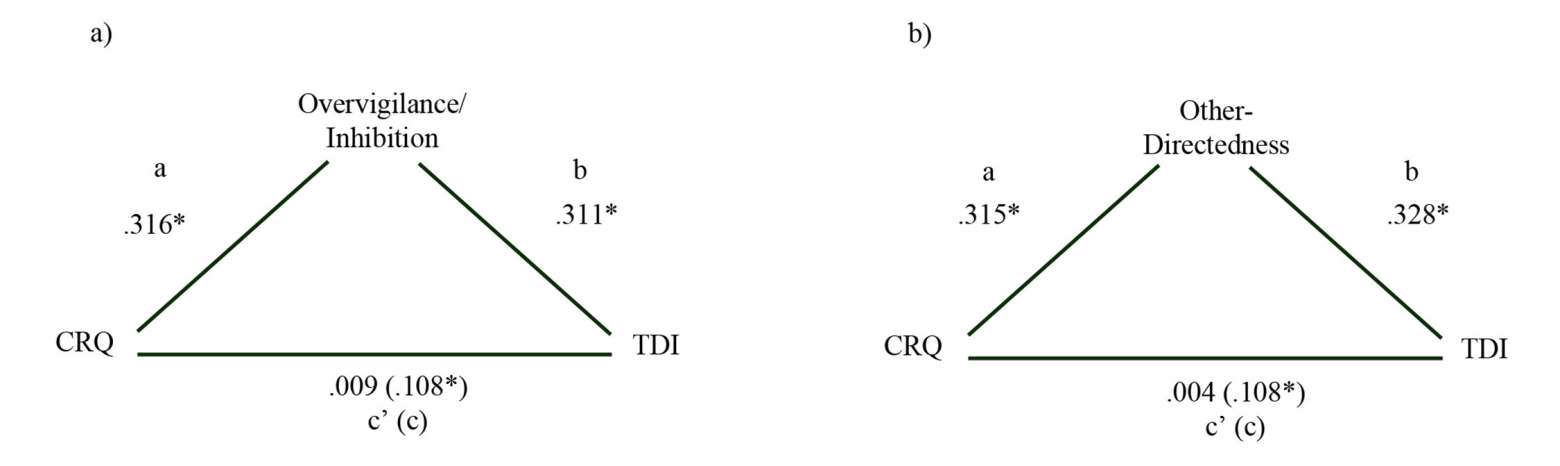
Mediation Diagrams with Standardized Coefficients. * *p* < .05. *Note*. CRQ = Co-Rumination Questionnaire; TDI = Teate Depression Inventory. Path *c* is the total effect, path *c’* represents the direct effect of co-rumination, a and b paths are the indirect effects.

To test whether each of the two schema domains mediated the relationship between co-rumination and depression, beta coefficients of three multiple regression equations were estimated and compared, following Baron and Kenny [42]. The path diagram of the selected mediation models is depicted in Fig 1.

These regressions showed that co-rumination was significantly associated with depression (*β* = .108, *t* = 2.325, *p* = .021) and Overvigilance/Inhibition (*β* = .316, *t* = 7.147, = .001) in the full study sample. After controlling for Overvigilance/Inhibition, the relationship between co-rumination and depression dropped dramatically to non-significant (*β* = .009; *t* = .201, *p* = .840), while the influence of Overvigilance/Inhibition remained significant. Therefore, Overvigilance/Inhibition was concluded to fully mediate the co-rumination-depression relationship (Fig 1A).

Co-rumination also was significantly associated with Other-Directedness (*β* = .108, *t* = 2.325, *p* = .021). After controlling for Other-Directedness, the relationship between co-rumination and depression decreased to non-significant (*β* = .004; *t* = .097, *p* = .923.). Thus, Other-Directedness was concluded to fully mediate the co-rumination-depression relationship (Fig 1B).

In order to test possible gender differences in the mediation effects exhibited in the total sample, these steps of mediation analysis were repeated separately for males and females. Results are presented in Table 2.

**Table 2:** Coefficients for paths in Mediation Analysis in male and female subsamples.

Step*	Path	Estimate	95% CI	Beta	*t*	*p*	Estimate	95% CI	Beta	*t*	*p*
*Overvigilance/ Inhibition*							*Other-Directedness*				
*Females*											
1	c	.094	.005 to .184	.130	2.083	.038	094	.005 to .184	.130	2.083	.038
2	a	.251	.160 to .342	.324	5.444	< .001	.224	.140 to .309	.312	5.219	< .001
3	b	.345	.230 to .459	.367	5.930	< .001	.328	.204 to .453	.325	5.193	< .001
4	c'	.008	-.081 to .097	.011	.176	.860	.021	-.069 to .110	.029	.456	.649
*Males*											
1	c	.022	-.093 to .138	.027	.380	.704	.022	-.093 to .138	.027	.380	.704
2	a	.316	.197 to .435	.344	5.242	< .001	.296	.171 to .421	.311	4.681	< .001
3	b	.288	.160 to .416	.315	4.426	< .001	.299	.178 to .420	.340	4.877	< .001
4	c'	-.069	-.186 to .049	-.082	-1.150	.251	-.066	-.182 to .049	-.079	-1.125	.258

*Baron and Kenny mediation steps method.

Estimate = standardized regression coefficient for specified path. Beta = unstandardized regression coefficient for specified path.

For females, the association of co-rumination and depression (path c) was statistically significant (*β* = .094, *t* = 2.083, *p* = .038). When controlling for Overvigilance/Inhibition, this relation dropped dramatically to not significant (*β* = .008; *t* = .176, *p* = .860). Similarly, this relation dropped to not significant when controlling for Other-Directedness (*β* = .021; *t* = .456, *p* = .649). Therefore, Overvigilance/Inhibition and Other-Directedness were concluded to fully mediate the co-rumination-depression relationship in the female subsample.

In the male subsample, the total effect from co-rumination to depression was not statistically significant (*β* = .022; *t* = .380, *p* = .704). However, in order to test the possibility of inconsistent mediation, the mediation analysis proceeded. When accounting for Overvigilance/Inhibition, the co-rumination-depression relationship became negative but remained not statistically significant (*β* = -.069; *t* = -1.150, *p* = .251). Similar results were found when controlling for Other-Directedness (*β* = -.066; *t* = -1.125, *p* = .258). Therefore, there was no evidence for any type of mediation, including inconsistent mediation, for Overvigilance/Inhibition and Other-Directedness in the co-rumination-depression relationship in the male subsample.

## Discussion

Although research related to the association of co-rumination and emotional distress is growing [1–6,47], little is known about the underlying processes of this relationship; that is, the mechanisms driving harmful effects of intensively discussing problems. The present study explored maladaptive cognitions as potential routes through which co-rumination may be associated with depression. This study appears to be the first to investigate the role that cognitive schema domains play in the co-rumination-depression relationship, as well as the first to use the YSQ, which affords researchers and clinicians the opportunity to explore maladaptive beliefs at a much deeper level than other available measures.

In this sample of Italian young adults, all five-schema domains were significantly associated with co-rumination. Among these, Overvigilance/Inhibition and Other-Directedness domains were not only significantly but also substantively related to co-rumination. A hypothetical model was tested in which these two schema domains were found to fully mediate the association between co-rumination and symptoms of depressive psychopathology in non-clinical young adults.

In order to interpret these findings, it is helpful to carefully consider the operational definitions of Overvigilance/Inhibition and Other-Directedness schemas in Young et al.’s [15] theory. Overvigilance/Inhibition is defined as “excessive emphasis on suppressing one’s spontaneous feelings, impulses and choices or on meeting rigid, internalized rules and expectations about performance and ethical behaviour, often at the expense of happiness, self-expression, relaxation, close relationships or health” (pp. 14–17) [15]. There are two specific types of schemas associated with this domain: Emotional Inhibition, which refers to excessive inhibition of spontaneous emotions or behaviors in order to avoid disapproval, shame, or a loss of impulse control; and Unrelenting Standards/Hypercriticalness, which refers to striving to meet the highest standards of behavior and performance in order to avoid criticism. Other-Directedness schemas involve “an excessive focus on the desires, feelings and responses of others at the expense of one’s own needs in order to gain love and approval, maintain one’s sense of connection or avoid retaliation” (p. 14–17) [15]. Other-Directedness schemas include: Subjugation, which is excessive surrendering of control to avoid negative consequences; and Self-sacrifice, which is the excessive focus on gaining approval, recognition, or attention from others in daily situations.

Thus taken together, cognitive schemas grouped in Overvigilance/Inhibition and Other-Directedness domains focus on meeting the needs of others at the expense of one’s own needs and well-being, and suppressing one’s emotional expression in the service of meeting high internalized standards and expectations about performance and ethical behavior. Moreover, according to Young and colleagues, these two domains correspond to the frustration of two basic psychological needs in childhood: playfulness and self-directedness, respectively [48].

The current findings may indicate that individuals prone to engage in co-ruminative discussions with their closest friend more easily activate a number of maladaptive schemas, particularly those related to subjugation, self-sacrifice, approval seeking, pessimism, emotional inhibition, hypercriticalness, and punitiveness—which, in turn, contribute to elevated levels of depressive symptoms. In fact, this process may elicit behaviors that perpetuate and/or reinforce these cognitive and affective experiences. For instance, Rose [1] provides the following illustrations of a co-ruminative coping style: ‘‘talking at length about whether the ambiguous behavior of a boyfriend or girlfriend is signaling the demise of the relationship or whether a perceived slight by a high-status classmate was intended or not” (p. 1830). Such discussions may trigger Other-Directed imaginings of threatened social disapproval in a relationship and subsequent efforts to increase closeness in the relationship in order to try to protect against abandonment. A discouraging response from the other person, or even the deterioration of the relationship, could easily reinforce the person's Other-Directedness schema, as well as lead to symptoms of depression.

It is interesting to note that the specific schema domains of Overvigilance/Inhibition and Other-Directedness fully mediated the association of co-rumination and depression. This is consistent with previous research investigating the specific topics of co-rumination that are problematic for adolescents and emerging adults, such as romantic experiences, social performance, and body-related concerns [6,7,11,12]. A closer look reveals that these co-ruminative topics involve excessive worrying for one’s social image and for one’s adaptation to social roles, which are specific cognitions that underlie the two domains of Overvigilance/Inhibition and Other-Directedness.

### Gender Differences in the Mediational Model

Our second, exploratory hypothesis concerned gender differences in co-rumination, cognitive schema domains, depression and/or the associations among these variables. Preliminary analyses revealed significant gender differences in the CRQ and TDI total scores, with females reporting higher levels of co-rumination and depressive symptoms than males. We re-conducted correlational analyses while splitting the total sample for gender of subjects. Similar to the relation between co-rumination and alcohol use [49], the relation between co-rumination and depression differed dramatically across genders. Among women, higher levels of co-rumination were associated with greater depression. In contrast, higher levels of co-rumination were not significantly associated with depression among men. Not surprisingly, this pattern suggests that the overall correlation between co-rumination and depression, found in the full sample, was driven entirely by the relationship between co-rumination and depressive symptoms in young adult women. These results suggest that co-rumination is more pervasive and may play a stronger role in depressive symptoms among young women compared to men, a finding that is consistent with previous research [2,5,50].

Thus, our proposed mediational model was supported only for females in this sample. It is possible that the lack of significant findings for males is driven by lower levels of co-rumination and/or depressive symptomatology among males. However, another intriguing possibility concerns differences in the *manner* in which females and males engage in co-rumination. To the extent that positive and negative facets of co-rumination might be isolated [9,51], it could be that the negative, dysfunctional, non-solution-focused component of co-rumination is more prevalent among young women compared to men. In other words, this type of co-rumination may be more common among females and may elicit maladaptive cognitive schemas which, in turn, lead to depressive outcomes. On the contrary, these schemas may remain inactive in co-ruminating males, and do not lead to emotional maladjustment.

Our results intimate that gender may actually moderate the relationship between co-rumination and depression in young adults, though this requires further empirical examination. Accordingly, important issues to be addressed, both theoretically and clinically in future studies, are whether the implicated early maladaptive schemas are specific to associations of co-rumination with depression only or also with other internalizing symptoms, such as anxiety.

### Limitations, future directions, and practical applications

This study suffered from a number of shortcomings. First, we focused only on Young’s [14] cognitive maladaptive schemas. Alternative constructs of dysfunctional cognitive processes, such as thought suppression, rumination, experiential avoidance, and perfectionism, could be involved in the association of co-rumination and depression. Future research on the role of maladaptive schemas could systematically expand on the present findings by incorporating additional or alternate operationalization of cognitive processes. Second, we examined gender as a factor influencing the co-rumination-depression relationship in a non-clinical young adult sample. Although this focus builds on research related to co-rumination and depression, future work may benefit from examining alternate individual difference variables such as religious group affiliations, cultural memberships, as well as other socio-demographic variables, such as marital status, age, and education [52,53]. Future work could examine these variables as potential moderators within the co-rumination-psychopathology association in youth. In addition to including other measures of maladaptive schemas, the current findings, based on Italian subjects, are in need of replication in non-clinical as well as clinical samples both domestically and internationally.

Third, the study is cross-sectional in nature, making it impossible to draw conclusions about cause-effect relationships. As all measures were concurrent, mediated relations should be interpreted in terms of indirect associations [54]. Although some evidence has been provided that co-rumination predicts prospective changes in youth’s depressive symptoms [5], the current study cannot rule out the possibility that causation flows from depression and/or maladaptive cognitions to co-rumination. That is, maladaptive cognitive schemas and/or depression may elicit co-rumination, or co-rumination may mediate the schema-depression relation. Future longitudinal and/or experimental studies are necessary to draw conclusions regarding the developmental trajectories of co-rumination, maladaptive cognitions, and internalizing symptoms. It should also be noted that recent studies have questioned Baron and Kenny’s casual steps method of mediation analysis [55]. Alternative contemporary approaches to mediation provide an overarching framework that considers two dimensions: the indirect effect and the direct effect, rather than “full or partial” classification employed by Baron and Kenny [56]. However, the use of one or another method depends on the conceptualization and measurement model that researcher prefers to test. The current investigation relied solely on paper-and-pencil self-report questionnaires to measure psychological constructs and on one individual’s perception of co-rumination. Future research should include other methods, such as observational procedures, psychophysiological assessment, or behavioral measures, as recommended elsewhere [57]. Co-rumination ideally should be assessed from the perspective of both members of the relationship dyad, given that the interpersonal impact of this process may vary between individuals within the same dyad [1]. Finally, selection bias may have contributed to our findings as over four-fifths of respondents were undergraduate students. The current results must be interpreted in light of this limit to external validity.

In spite of these limitations, the present findings have both theoretical and applied value. Better understanding of the processes underpinning co-rumination can inform depression prevention and intervention programs. These data suggest that Schema Therapy [15,58], an integrative treatment targeting the reduction of maladaptive cognitive schemas and the activation of adaptive schemas, may enable young adults to respond to challenges by selecting alternative coping mechanisms to co-rumination. Young adults who report high levels of co-rumination also might benefit from therapies addressing core beliefs, which could help them increase the range of their coping strategies. By adaptively using their own social resources, young adults may be able to thwart the onset of core beliefs in depression such as helplessness, hopelessness, and inadequacy. Research has demonstrated that symptom relief is predicted by the degree of change in early maladaptive schemas over the course of Schema Therapy [59].

Given the different levels and implications of co-rumination for young women versus men, gender-specific intervention and prevention strategies might be particularly effective. For instance, prevention programs for girls may need to target a reduction in the extent of co-rumination by teaching strategies to minimize an exclusive focus on negative topics in discussions or alternate activities with talking [4,60].

Finally, these findings may be useful for educators and college students. It is well known that co-rumination interrupts normal, positive problem-solving strategies, and though it can feel comforting, it can exact high emotional and intellectual costs for college students. At the University of Richmond, a co-rumination campaign has been undertaken [19]. Residence life educators have applied knowledge about co-rumination to their work with dissatisfied students who are contemplating transferring out of their institution. Through a series of lessons, one-on-one discussions and role-plays, interventionists have supported students in replacing co-ruminative habits with more adaptive strategies for coping with college stressors. Effects of this campaign have been striking. Findings of the present study suggest the potential utility of incorporating a set of Schema Therapy sessions into such programs. Interventions that target this sequence of maladaptive responses could short-circuit the unfolding of co-ruminative patterns and thereby promote adolescents’ and young adults’ emotional, intellectual, and social well-being.

## Supporting Information

S1 File Dataset. (SAV)
